# Comparison of nasogastric tube and intermittent oro-esophageal tube feeding in patients with dysphagia and evaluation of an assessment scale for initial nutritional support selection

**DOI:** 10.3389/fnut.2026.1839716

**Published:** 2026-06-23

**Authors:** Yu-Long Zhao, Gui-Wei Lu, Zhen-Qing Ren, Yan-Hua Xu, Yu Gao, Jin-Xia Sun

**Affiliations:** 1 Department of Anesthesiology, The Affiliated Taizhou People’s Hospital of Nanjing Medical University, Taizhou, China; 2 Department of Rehabilitation, The Affiliated Taizhou People’s Hospital of Nanjing Medical University, Taizhou, China; 3 Department of Nursing, The Affiliated Taizhou People’s Hospital of Nanjing Medical University, Taizhou, China

**Keywords:** assessment scale, dysphagia, initial, intermittent oro-esophageal tube feeding, nasogastric tube feeding, nutritional support method

## Abstract

**Objective:**

The aim of this study was to assess the clinical outcomes of nasogastric tube (NGT) feeding and intermittent oro-esophageal tube (IOE) feeding in patients with moderate-to-severe dysphagia, and to evaluate the IOE assessment scale in guiding the initial selection of nutritional support strategies.

**Methods:**

A total of 208 patients with stroke complicated by moderate-to-severe dysphagia (Chinese version of the Volume and Viscosity Swallowing Test, VVST-CV ≥ moderate impairment) who were admitted to the Cerebrovascular Center of Taizhou People’s Hospital Affiliated to Nanjing Medical University between February 2024 and April 2025 were enrolled. Among these patients, 148 met the indications for IOE according to the IOE assessment scale. Of these, 124 patients with a hospital stay of ≥ 28 days were allocated into two groups based on the results of the IOE assessment scale: the NGT group (*n* = 64, control group) and the IOE group (*n* = 60, study group). Improvements in swallowing function, changes in the feeding component of activities of daily living (ADL), nutritional status and fluid intake, comfort level, and the incidence of tube dislodgement and aspiration were compared between the two groups.

**Results:**

Improvement in swallowing function was significantly greater in the IOE group than in the NGT group (*p* < 0.01). The increase in ADL feeding scores was more pronounced in the IOE group. On day 28, mean serum albumin score were significantly higher in the IOE group than in the control group (*p* < 0.01). Total fluid intake from day 1 to day 28 was significantly higher in the IOE group (*p* < 0.01). Patients in the IOE group reported higher comfort levels, and the incidences of aspiration and tube dislodgement were significantly lower compared with the NGT group (*p* < 0.05).

**Conclusion:**

The IOE assessment scale may help guide the selection of nutritional support strategies for patients with dysphagia. In this study, compared with NGT, IOE feeding was associated with a shorter recovery time of swallowing function, improved physical and psychological comfort, enhanced nutritional status, increased fluid intake, and reduced risks of aspiration and tube dislodgement.

## 1 Introduction

Dysphagia is a common complication following stroke and is frequently observed in older patients with neurological disorders. It is associated with an increased risk of malnutrition, pneumonia, and mortality (1, 2). Currently, nutritional support for patients with dysphagia is provided through nasogastric tube (NGT) feeding or gastrostomy in both China and other countries. However, prior studies have reported that long-term indwelling nasogastric or nasoenteric tubes are associated with various complications, including nasogastric tube syndrome, pulmonary infection, chronic sinusitis, gastroesophageal reflux, and unplanned tube removal (3). Additionally, discomfort related to prolonged indwelling nasogastric tubes may reduce patient adherence to rehabilitation training, which may adversely affect the recovery of swallowing function. Gastrostomy is an invasive procedure that carries a risk of postoperative infection, and acceptance of this approach remains limited among many patients in China. With ongoing advancements in rehabilitation techniques and increasing expectations among patients regarding treatment effectiveness, safety, comfort, and psychological well-being during hospitalization, there is a need to examine improved methods and technologies for nutritional support.

Intermittent oro-esophageal tube (IOE) feeding is a method of enteral nutritional support. This technique involves inserting a tube through the oral cavity into the esophagus for feeding, with removal immediately after completion of feeding (4). The procedure is relatively simple and does not require continuous involvement of medical staff, as it may be performed independently by patients or their family members. It is generally well tolerated and is widely accepted by patients and their families. Therefore, it is particularly suitable for patients with stroke complicated by dysphagia. In clinical practice, the selection of an appropriate tube-feeding modality for nutritional support remains a key consideration and constitutes the focus of this study.

The design of the IOE assessment scale is presented in Table 1. Targeted evaluation using the items of this scale facilitates selection of a tube-feeding modality that is more effective, more comfortable, and conducive to recovery of swallowing function in patients with dysphagia (Chinese version of the Volume and Viscosity Swallowing Test (VVST-CV) ≥ moderate impairment) (5). This approach contributed to improved clinical safety, enhanced patient comfort, improved activities of daily living (ADL), and promotion of recovery of swallowing function.

**Table 1:** IOE assessment scale for dysphagia.

Recording time: Name: Age: Hospital ID: VVST-CV:					
Purpose: To provide guidance for selecting an appropriate method of nutritional support for patients with dysphagia.					
A. Write the number corresponding to each option in the box. 0 = none, 1 = mild, 2 = moderate, 3 = severe					
1	Consciousness	Alert	Confused	Stuporous	Deep coma
		0	1	2	3
2	Muscle strength of unaffected upper limb	Grade 5	Grade 4	Grade 3	Grade 1–2
		0	1	2	3
3	Joint mobility of unaffected upper limb	Grade 5	Grade 4	Grade 3	Grade 1–2
		0	1	2	3
4	Daily frequency of food and fluid intake	3–6 times	7–8 times	9–10 times	Continuous enteral pump ≥15 h
		0	1	2	3
5	Social support system	Excellent	Good	Fair	Poor
		0	1	2	3
B. Total score					
C. Results and recommendations: If any item scores ≥ 2, IOE is not recommended for nutritional support. IOE is contraindicated in patients with pharyngeal or esophageal injury.					
D. 1. Intermittent oro-esophageal tube (IOE) feeding involves inserting a tube orally into the esophagus only during feeding, without leaving the tube in the stomach. The tube is removed after each feeding, and patients receive necessary nutrients according to their usual feeding patterns.					

Therefore, a total of 208 patients with stroke complicated by dysphagia (VVST-CV ≥ moderate impairment) who were treated at the Cerebrovascular Center of the Affiliated Taizhou People’s Hospital of Nanjing Medical University between February 2024 and April 2025 were enrolled. Following assessment with the IOE assessment scale, 148 patients met the criteria for nutritional support via IOE. Among these, 124 patients with a hospital stay of ≥ 28 days were included in the prospective analysis.

## 2 Data and methods

### 2.1 General data

A total of 208 patients with post-stroke dysphagia (VVST-CV ≥ moderate impairment) who were admitted to the hospital were selected. Following evaluation using the IOE assessment scale (Table 1), 148 patients met the criteria for nutritional support via IOE. Among these, 124 patients with a hospital stay of ≥ 28 days were included. The IOE assessment scale was used solely to screen for eligibility (i.e., to identify patients who were medically suitable for IOE feeding). After the scale assessment, all eligible patients and their family members received standardized education on both NGT and IOE feeding, including procedures, benefits, risks and daily management. Patients or their legal guardians then made a voluntary choice regarding which feeding method to receive. This approach was adopted because NGT feeding is the conventional method widely accepted by patients at our institution, whereas IOE feeding is a newer technique that many patients are unfamiliar with. Respecting patient autonomy was considered ethically essential. Consequently, among the 124 eligible patients, 64 chose NGT (control group) and 60 chose IOE (study group). No patient received a feeding method that was different from their own choice. Importantly, because the IOE assessment scale only provides a classification of “suitable for IOE or not,” and does not recommend against NGT for eligible patients, there was no case where a patient received a method inconsistent with the scale’s recommendation. The NGT group was designated as the control group, and the IOE group was designated as the study group. During the study period, 4 patients in the control group withdrew, and 60 patients remained in the control group for final analysis. In the study group, 2 patients withdrew, and 58 patients remained for final analysis. Comparison of baseline characteristics between the two groups indicated no statistically significant differences (*p* > 0.05), indicating baseline comparability (Table 2).

**Table 2:** Baseline characteristics of patients.

Group	Cases	Male/Female (*n*)	Age (x̄ ± s, years)	Intracerebral hemorrhage/Cerebral infarction (*n*)	VVST-CV (Moderate/Severe) (*n*)
Control group	60	31/29	61.55 ± 11.70	37/23	47/13
Study group	58	31/27	61.36 ± 15.25	35/23	45/13
χ^2^/*t* value	-	0.038	0.075	0.022	0.010
*p*-value	-	0.85	0.94	0.88	0.92

### 2.2 Inclusion and exclusion criteria

Inclusion criteria: Patients who met the diagnostic criteria for stroke; patients diagnosed with swallowing dysfunction of moderate or greater severity (VVST-CV ≥ moderate impairment); patients with a hospital stay in the department of ≥ 28 days; patients without significant oral, pharyngeal, or esophageal disease; patients aged 18–85 years; and patients who provided informed consent or whose informed consent was provided by their legal guardians.

Exclusion criteria: Patients with unstable clinical conditions; patients with recent severe upper gastrointestinal discomfort; patients with a hospital stay in the department of < 28 days; patients with a history of nasopharyngeal carcinoma or laryngeal tumor treated with surgery or radiotherapy; patients with a history of gastric varices; patients who declined participation; and patients unable to cooperate due to cognitive impairment.

### 2.3 Methods

Routine swallowing function training was administered to all patients in both groups after enrollment. Prior to the resumption of oral intake in either group, all meals were uniformly prepared and supplied by the Nutrition Department under dietitian supervision to ensure nutritional consistency and comparability between groups.

In the control group, nutritional support was provided via NGT. The procedure was performed with the patient in a sitting position or with the head of the bed elevated to 30°. A 14-Fr nasogastric tube was inserted through the nasal cavity into the stomach and secured in place. The tube was maintained indwelling. Enteral nutrition suspension (500 mL per bottle) was administered into the stomach using a nutrition pump at a rate of 90–110 mL/h, with 2–3 bottles provided per day. After each feeding, the nasogastric tube was flushed with 20–40 mL of warm water.

In the study group, nutritional support was provided via IOE. The procedure was conducted as follows (6):

1. Preparation before feeding: The patient was assisted into a sitting or semi-sitting position, and dentures were removed. An 8–12 Fr silicone gastric tube was selected and lubricated with warm water or paraffin oil. The anterior segment of the tube was naturally curved.
2. Tube insertion: The patient was instructed to open the mouth. The gastric tube was advanced along a parabolic trajectory, maintaining close contact with the posterior wall of the oral cavity on the side affected by oral numbness or swallowing paralysis. During passage through the pharynx, the patient was instructed to swallow slowly to facilitate advancement of the tube through the esophagus into the stomach. After confirming that the gastric tube is positioned in the stomach, withdraw the distal end of the tube to the upper esophagus (total insertion length approximately 25–30 cm).
3. Criteria for successful placement: Absence of coughing and stable respiration were observed; the distal end of the tube was submerged in water without the presence of escaping bubbles.
4. Feeding: Food, water, and medication were delivered through the tube into the upper esophagus. The feeding volume per meal was determined according to daily requirements, generally involving 4–6 insertions per day, administered at 38–40 °C, with 300–500 mL per feeding.
5. Post-feeding procedure: After feeding, the distal end of the tube was folded. The patient was instructed to take a deep breath, and the tube was removed rapidly at the end of exhalation. The patient was instructed to maintain the position for approximately 30 min.
6. Tube care: The removed tube was rinsed with warm water and dried for reuse.
7. Single-use tubes were replaced 2–3 times per week, and silicone tubes were replaced once per week.
8. Training and guidance: The first IOE procedure was conducted by clinical staff, and the procedure and precautions were explained to the patient and family members. Subsequent insertions were performed by the patient under supervision, with assistance provided by family members as needed.

The IOE assessment scale was developed as a screening tool to guide the selection of nutritional support modalities in patients with dysphagia and to facilitate its exploratory clinical application. The scale is designed based on factors relevant to a patient’s ability to complete the feeding process, including level of consciousness, muscle strength of the unaffected upper limb, joint mobility of the unaffected upper limb, daily frequency of nutritional and fluid intake, and social support system. These items were derived from extensive clinical observation and summarization of patients with dysphagia. A deficiency or insufficiency in any item (reflected by a higher score) would compromise the patient’s suitability for IOE. In the present study, the scale was used exclusively as an eligibility screening tool to identify patients medically suitable for IOE feeding. It did not automatically assign patients to the IOE group; instead, final feeding modality was determined by patient/family preference after receiving standardized information about both methods. This design reflected real-world clinical decision-making while maintaining safety screening.

Data were collected for both the control and study groups at admission and at weekly intervals from week 1 to week 4. Collected variables included the results of the VVST-CV, the feeding component of the ADL scale, nutritional status, comfort level, and the incidence of aspiration and tube dislodgement.

### 2.4 Observation criteria

At the same time points described above, comparisons between the two groups were conducted with respect to improvement in swallowing function, the rate of improvement in the feeding component of ADL, nutritional status and fluid intake, physical comfort, and the incidence of aspiration and tube dislodgement.

Improvement in swallowing function was assessed based on VVST-CV results. An increase of 2–3 grades compared with baseline was defined as marked improvement; an increase of 1 grade was defined as improvement; and no change was defined as no improvement. The effective rate of swallowing function improvement was calculated as follows: Effective rate = (marked improvement + improvement) / total cases × 100% (7).

Improvement in ADL was assessed using the Modified Barthel Index, with specific assessment of the feeding component (8). Scoring criteria were defined as follows: complete dependence = 0 points; major assistance = 2 points; moderate assistance = 5 points; minimal assistance = 8 points; and complete independence = 10 points. An increase of 2–3 grades compared with baseline was defined as marked improvement; an increase of 1 grade was defined as improvement; and no change was defined as no improvement.

Nutritional status was assessed using the Nutritional Risk Screening 2002 (9). Serum albumin–based scoring was defined as follows: ≥40 g/L = 0 points; 35–39 g/L = 1 point; 30–34 g/L = 2 points; and <30 g/L = 3 points. The average daily fluid intake over 28 days was recorded for both groups.

Comfort was assessed using a standardized four-point ordinal comfort scale adapted from the comfort care model validated in Chinese patients with indwelling nasogastric and tracheal tubes (10): The scale evaluates oral and pharyngeal discomfort across four dimensions: mucosal dryness, pain, sputum accumulation, and signs of mucosal inflammation. Grade I indicated no obvious discomfort (1 point); Grade II indicated mild discomfort, characterized by dryness of the oral cavity and pharynx (2 points); Grade III indicated moderate discomfort, characterized by dryness, pain, or the presence of phlegm in the oral cavity and pharynx (3 points); and Grade IV indicated severe discomfort, characterized by oral inflammation, excessive phlegm, or similar manifestations (4 points). Assessments were performed consistently by trained nursing staff at each time point. Of note, formal reliability and validity testing of this scale in dysphagia populations has not yet been conducted.

The incidence of aspiration and tube dislodgement was compared between the groups. Aspiration data were collected only for events occurring during NGT or IOE feeding, and episodes of aspiration that occurred prior to tube placement were excluded.

### 2.5 Statistical methods

Statistical analyses were conducted using SPSS 26.0. Continuous variables that conformed to a normal distribution were expressed as mean ± standard deviation (x̄ ± s) and were compared between groups using the *t*-test. Categorical variables were expressed as the number of cases (*n*) and percentage (%) and were analyzed using the chi-squared (χ^2^) test. The effects of the interventions on the observed indicators and their changes over time were assessed using two-way repeated-measures analysis of variance. A *p* value < 0.05 was considered statistically significant, and *p* < 0.01 was considered highly statistically significant. This study did not perform a formal *a priori* sample size calculation due to its exploratory nature and the practical constraints of patient enrollment within the study period. The sample size was determined by the number of eligible patients who met the inclusion criteria during the recruitment window. Future confirmatory studies with adequately powered sample sizes are warranted.

## 3 Results

### 3.1 Comparison of swallowing function improvement between the control and study groups

Swallowing function was assessed at admission and from day 1 to day 28. In the control group, the numbers of patients classified as having marked improvement, improvement, and no improvement were 0, 9, and 51, respectively, yielding an effective rate of 15%. In the study group, the corresponding numbers were 3, 30, and 25, respectively, with an effective rate of 56.9%. The difference between the two groups was statistically significant (χ^2^ = 23.175, *p* < 0.01).

### 3.2 Comparison of ADL feeding item scores

As presented in Table 3, ADL feeding item scores were recorded at admission and from day 1 to day 28. In the control group, the mean scores were 0, 0, 0.08, 0.3, and 0.8, respectively. In the study group, the corresponding mean scores were 0, 1.17, 1.88, 3.16, and 4.59. Nutritional support delivered via IOE demonstrated a statistically significant effect on improvement in ADL feeding item scores [*F*(1, 116) = 50.82, *p* < 0.0001], reflecting enhanced feeding ability among patients.

**Table 3:** ADL feeding item scores in the two groups.

Group	At admission	After admission			
		7d	14d	21d	28d
Control Group	0.00 ± 0.00	0.00 ± 0.00	0.083 ± 0.64	0.30 ± 1.35	0.80 ± 2.21
Study Group	0.00 ± 0.00	1.17 ± 1.68**	1.87 ± 2.30****	3.15 ± 2.91****	4.59 ± 3.69****

Compared with the control group: *p < 0.05, **p < 0.01, ***p < 0.001, ****p < 0.0001.

### 3.3 Comparison of nutritional status and fluid intake between the control and study groups

At admission, the mean serum albumin score was 0.3 in the control group and 0.31 in the study group, with no statistically significant difference between the groups (*p* > 0.05). On day 28, the mean serum albumin score in the control group was 0.87, and the average fluid intake from day 1 to day 28 was 1,891.67 mL. In the study group, the corresponding mean serum albumin score was 0.22, and the average fluid intake was 2,189.40 mL. The difference in mean serum albumin scores on day 28 between the two groups was statistically significant (*t* = 6.508, *p* < 0.01). The difference in average total fluid intake from day 1 to day 28 between the control and study groups was statistically significant (*t* = 3.084, *p* < 0.01).

### 3.4 Comparison of comfort scores

Comfort data were collected at admission and from day 1 to day 28 during NGT and IOE feeding. In the control group, the mean comfort scores were 1.62, 1.72, 1.97, 2.1, and 2.22, respectively. In the study group, the corresponding mean scores were 1.29, 1.17, 1.14, 1.14, and 1.17. Nutritional support delivered via IOE was associated with a statistically significant improvement in comfort scores [*F*(1, 116) = 60.86, *p* < 0.0001]. Detailed data are presented in Table 4.

**Table 4:** Comfort scores in the two groups.

Group	At admission	After admission			
		7d	14d	21d	28d
Control Group	1.62 ± 0.6	1.72 ± 0.63	1.97 ± 0.75	2.10 ± 0.83	2.22 ± 0.84
Study Group	1.30 ± 0.46*	1.18 ± 0.38****	1.14 ± 0.34****	1.14 ± 0.39****	1.18 ± 0.50****

Compared with the control group: *p < 0.05, **p < 0.01, ***p < 0.001, ****p < 0.0001.

### 3.5 Comparison of tube dislodgement and aspiration between the two groups

During the 28-day observation period, 4 cases of accidental tube dislodgement and 3 cases of aspiration were recorded in the control group. In the study group, no cases of accidental tube dislodgement and 1 case of aspiration were observed. The differences between the two groups were statistically significant (χ^2^ = 4.613, *p* < 0.05).

## 4 Discussion

Dysphagia is a common complication of stroke that impairs oral intake of food and fluids, adversely affects nutrient digestion and absorption, prolongs hospitalization, and increases medical costs. In severe cases, it may result in death (11). Dysphagia is an important clinical manifestation, and its incidence increases with advancing age (12). Therefore, selection of an appropriate method for nutritional and fluid intake in patients with dysphagia is essential. An appropriate feeding strategy may reduce the risk of aspiration, promote recovery of swallowing function, improve ADL, and enhance patient comfort. These issues represent important considerations in clinical management. Based on the findings of this study, IOE demonstrated several advantages compared with NGT:

IOE was characterized by relatively fixed mealtimes, with tube insertion performed immediately prior to feeding and removal completed after feeding. This approach was consistent with the physiological and psychological patterns associated with oral intake, was associated with less discomfort, and was conducive to improved patient adherence. The volume of food and water administered per meal more closely approximated habitual intake. A randomized controlled trial conducted in healthy individuals comparing intermittent high-volume feeding with continuous enteral nutrition demonstrated that intermittent high-volume feeding increased mesenteric arterial blood flow, elevated insulin and peptide YY levels, and reduced blood glucose concentrations (13). Additionally, IOE provided repeated pharyngeal stimulation, which may contribute to improvement in swallowing function, enhancement of respiratory coordination, and reduction in the incidence of aspiration (14). Improvement in swallowing function may shorten the duration of tube feeding. In the present study, comparison of swallowing function improvement between the control and study groups indicated a statistically significant difference (*p* < 0.01).

The initial IOE procedure was performed by clinical staff, during which the operational steps and precautions were explained to patients and their family members. Subsequently, patients were guided to perform oral tube insertion independently, with assistance from family members when necessary. After mastering the procedure, they could carry out the technique independently, thereby promoting active participation in rehabilitation and contributing to improved quality of life. In this study, comparison of the ADL feeding component between the control and study groups demonstrated that IOE was superior to NGT. The difference in ADL feeding scores between the experimental and control groups increased with longer durations of hospitalization.

Malnutrition represents a significant clinical concern that may exacerbate the condition of patients with stroke and contribute to adverse outcomes. Dysphagia is a major contributing factor to malnutrition (15). Following improvement in swallowing function, patients receiving IOE were able to consume a broader range of foods and nutrients. The study demonstrated that on day 28, the mean serum albumin score in the study group was significantly lower than that in the control group, with a statistically significant difference (*p* < 0.01). The difference in average total fluid intake from day 1 to day 28 between the control and study groups was statistically significant (*p* < 0.01). It should be noted that serum albumin levels are influenced by factors beyond nutritional intake, including systemic inflammation, hydration status, and overall disease severity. Therefore, the observed improvement in serum albumin scores in the IOE group may reflect not only enhanced nutritional delivery and the earlier resumption of oral intake, but also the broader clinical benefits of IOE, such as improved swallowing function and reduced rates of aspiration and infection. These contributing factors cannot be fully disentangled in the present study.

Patients receiving IOE remained without an indwelling tube for most of the time, which was associated with improved comfort and reduced impact on self-image. In this study, IOE demonstrated superiority over NGT with respect to comfort, and the difference between the experimental and control groups became more pronounced with increasing duration of hospitalization.

Since the IOE tube was not retained in the stomach for prolonged periods, interference with lower esophageal sphincter function was avoided, and the nasopharyngeal cavity remained unobstructed. Comparison of accidental tube dislodgement and aspiration between the control and study groups demonstrated statistically significant differences (*p* < 0.05). During the period of tube use, 4 cases of accidental tube dislodgement and 3 cases of aspiration were observed in the control group, whereas no cases of tube dislodgement and 1 case of aspiration were recorded in the study group. These findings indicate that IOE was associated with a higher level of safety compared with NGT. The safety and feasibility of repeated IOE insertion by patients or their caregivers warrant specific consideration. In this study, a structured training protocol was implemented: clinical staff performed the initial insertion while explaining the procedure and precautions, and subsequent insertions were carried out by the patient under supervision, with caregiver assistance as needed. Several safeguards contributed to procedural safety: correct tube placement was confirmed by the absence of coughing, stable respiration, and the bubble test; the insertion depth was standardized at 25–30 cm to target the upper esophagus; and patients maintained a sitting or semi-sitting position for approximately 30 min after feeding. The low observed rates of aspiration (1 case) and tube dislodgement (0 cases) in the IOE group provide preliminary evidence supporting the safety of this approach when performed by appropriately trained individuals. Nevertheless, the feasibility of this training model in routine clinical practice, particularly in settings with limited staffing or resources, requires further evaluation. Standardized training materials, demonstration videos, and competency checklists would facilitate broader implementation.

An increasing number of studies have emphasized the limitations associated with long-term NGT (16). The incidence of pneumonia in patients receiving prolonged NGT has been reported to range from 31.25 to 70.00% (17). However, NGT remains widely used in most hospitals in China. In particular, NGT is routinely used in primary and secondary hospitals for the management and care of patients with moderate-to-severe dysphagia. Although IOE provides notable advantages for patients with swallowing disorders, its clinical application remains limited. One of the primary objectives of this study is to establish IOE screening method for clinicians, which is also hoped to lay the foundation for the development of an IOE assessment scale for patients with dysphagia.

From extensive clinical observation and systematic assessment of patients with dysphagia, the IOE assessment scale was developed. The scale assesses five domains: level of consciousness, muscle strength of the unaffected upper limb, joint mobility of the unaffected upper limb, daily frequency of nutritional and fluid intake, and status of the social support system to determine the suitability of IOE for individual patients. The IOE assessment scale was developed to provide an exploratory clinical application for selecting the initial tube-feeding modality for nutritional support in patients with dysphagia. This study was designed to promote the exploratory application of the IOE assessment scale in clinical practice, thereby assisting clinicians in making better choices between IOE tube feeding and PNG feeding.

However, the study has several limitations. The first limitation of this study is its non-randomized design, which was a methodological necessity to evaluate the scale’s clinical utility. The patient grouping was driven by the scale’s assessment, and this may have introduced selection bias. Future randomized controlled studies are warranted to validate the superiority of IOE over NGT, independent of the selection criteria. Second, while both groups received dietitian-prescribed standardized nutrition during the period of complete tube-feeding dependence, detailed daily records of actual caloric and protein intake were not available. The contribution of differential nutrient delivery during the transitional feeding phase cannot be fully quantified. Future studies should incorporate rigorous monitoring of nutritional intake to more precisely isolate the effects of feeding route from those of nutrient delivery. Third, with regard to statistical methodology, parametric tests were applied to several ordinal outcomes. Although this approach is common in rehabilitation research and was supported by acceptable distributional properties in our sample, non-parametric methods or ordinal regression models would provide greater statistical rigor and should be considered in future studies. Additionally, the non-randomized design precluded covariate-adjusted analyses; while baseline comparability was confirmed, the possibility of residual confounding cannot be entirely ruled out. Fourth, this was a single-center study with a relatively modest sample size, which may limit the generalizability of the findings to other clinical settings and populations, and also limit statistical power for detecting differences in rare adverse events. The 28-day observation period, while sufficient to capture early recovery of swallowing function, precludes assessment of longer-term outcomes and the durability of the observed benefits. In addition, the safety assessment was confined to aspiration events occurring during tube feeding and accidental tube dislodgement. Other clinically relevant adverse events, such as sinusitis, gastroesophageal reflux, and tube-related mucosal injury, were not systematically recorded. A more comprehensive safety profile remains to be established. Finally, the IOE assessment scale, although developed from extensive clinical observation, has not yet undergone formal validation (e.g., reliability testing, construct validity analysis, and inter-rater agreement assessment). The present study represents an initial step in its clinical application, and the scale requires further psychometric evaluation and external validation before it can be recommended for widespread use. Future randomized controlled trials with larger samples and pre-planned adjusted analyses are warranted.

## Data Availability

The original contributions presented in the study are included in the article/supplementary material, further inquiries can be directed to the corresponding author.
